# VEGFR2 promotes tumorigenesis and metastasis in a pro-angiogenic-independent way in gastric cancer

**DOI:** 10.1186/s12885-019-5322-0

**Published:** 2019-02-28

**Authors:** Lian Lian, Xiang-Li Li, Meng-Dan Xu, Xian-Min Li, Meng-Yao Wu, Yan Zhang, Min Tao, Wei Li, Xiao-Ming Shen, Chong Zhou, Min Jiang

**Affiliations:** 1 Department of Oncology, Suzhou Xiangcheng People’s Hospital, Suzhou, 215131 China; 2 Department of Oncology, the First Affiliated Hospital of Soochow University, Suzhou, 215006 China; 3 Department of General Surgery, Suzhou Xiangcheng People’s Hospital, Suzhou, 215131 China; 4 Comprehensive Cancer Center, Suzhou Xiangcheng People’s Hospital, Suzhou, 215131 China; 5 Department of Radiation Oncology, Xuzhou Central Hospital, Xuzhou, 221009 China

**Keywords:** Gastric cancer, VEGFR2, VTN

## Abstract

**Background:**

VEGF/VEGFR2 pathway is the central therapeutic target in anti-angiogenic treatment in multiple cancers. However, little work has been carried out concerning the pro-malignancy functions of VEGFR2 that are independent of its pro-angiogenesis effects in gastric cancer. Here, we demonstrated that VEGFR2 up-regulation in gastric cancer tissues was a prognostic marker for poor disease-free survival and overall survival of gastric cancer patients.

**Methods:**

Immunohistochemistry was used to detect VEGFR2 and VTN expressions in specimens. Kaplan–Meier curves were constructed for survival analysis. Stably knockdown cell lines and overexpression cell lines were constructed by small interfering RNA and plasmids transfection. Real-time PCR and Western blot were used to confirm the expressions of target genes at both RNA and protein levels. Cell proliferation was measured by using Cell Counting Kit-8 and xenograft models. Microarray and bioinformatic analysis were also performed to identify the relationship between Vitronectin (VTN) and VEGFR2.

**Results:**

When overexpressed in gastric cancer cells, VEGFR2 increased cellular proliferation and invasion in vitro and tumor formation in xenograft models. By using integrating microarray and bioinformatic analysis, we identifiedVTN as a downstream of VEGFR2 pathway. In gain- and loss-of function analysis in gastric cancer cells, VTN was further verified in consistent with VEGFR2 in expression levels and in regulating cell growth and motility in vitro and in vivo*.* Moreover, in gastric cancer samples, VTN was as also revealed as a poor prognostic factor.

**Conclusions:**

Our present findings defined a novel activity for VEGFR2 in promoting tumorogenicity, motility and indicating a poor survival in gastric cancer beyond its known pro-angiogenic effects.

**Implications:**

Our present findings defined a novel activity for VEGFR2 in promoting tumorogenicity, motility and indicating a poor survival in gastric cancer beyond its known pro-angiogenic effects, which may provide a new and valuable target for design of therapies for intervention and a new cognitive perspective for the anti-angiogenesis therapies.

**Electronic supplementary material:**

The online version of this article (10.1186/s12885-019-5322-0) contains supplementary material, which is available to authorized users.

## Introduction

VEGFR2 (Vascular endothelial growth factor receptor 2) is one of the two tyrosine kinase receptors involved in angiogenesis. When activated by its ligand VEGF, VEGFR2 promotes neighbouring vessel formation to facilitate the delivery of growth factors, nutrients and oxygen for cancer proliferation, migration, metastasis and survival [1]. VEGF and VEGFR2 mediated angiogenesis contributes to the aggressive natures and leads to high mortality rate in gastric cancer, which is the third leading cause of cancer deaths worldwide [2]. In patients with advanced gastric cancer, pharmacologic agents that specifically targeting VEGF ligand, or receptors by specific kinase inhibitors or antibodies, exhibited efficacy in clinical trials in combination with chemotherapy or not [3]. Of note, monoclonal antibody ramucirumab and tyrosine kinase inhibitor apatinib, both targeting VEGFR2, showed more favourable benefit compared to direct block of angiogenesis stimuli by bevacizumab [4], implying the complicated implication of VEGFR2 involved in tumorigenesis and progression of gastric cancer.

Over the past decades, besides of initiation of angiogenesis of VEGFR2 signaling in different malignancies, the lesser known direct impact of which on cancer cells cannot be overlooked [5]. In addition to its constitutive expression on endothelial cells, presence of VEGFR2 on cancer cells was also confirmed in breast cancer [6, 7] [8, 9], lung cancer [10], glioblastoma [11], gastrointestinal cancer [12], hepatocellular carcinoma, renal cell carcinoma [13], ovarian cancer [14], bladder cancer [15], and osteosarcoma [16]. Besides of the contribution of VEGFR2 that leads to tumor neovascularity in peri-cancer cellular niche, how or whether this proangiogenic factor receptor and its regulatory axis could regulate survival, malignant progression and invasion of gastric cancer, independent of VEGF-induced angiogenesis, is still not clearly understood. In the present study, we investigated the angiogenesis independent pro-malignancy function of VEGFR2 signaling in gastric cancer cells. These results may provide novel insights of VEGFR2 inhibitors in cancer cell level and new anticancer strategies for management of gastric cancer.

## Methods and materials

### Patients and samples

The experimental protocol was approved by the Human Ethics Committee of the Xiangcheng People’s Hospital and the First Affiliated Hospital of Soochow University. Written informed consent was collected from individual patient. A total of 156 surgical cancer specimens were obtained from patients with gastric cancer.

### Immunohistochemistry

All resection specimens in this study were fixed in 10% buffered formalin and paraffin embedded by routinely processing. Sections were cut at a thickness of 4 μm, heated at 60 °C for 30 min, then deparaffinized and hydrated through a series of xylene and alcohol baths before staining. The slides were microwaved with antigen retrieval solution (citrate buffer, pH 6.0, containing 0.3% trisodium citrate and 0.04% citric acid) for 5 min. After replenishment of this solution, the slides were microwaved again for an additional 5 min and then allowed to cool for 20 min. The sections were then rinsed in PBS (phosphate-buffered saline), and immersed in 3% H_2_O_2_ for 15 min to block the endogenous peroxidase. Thereafter, the sections were incubated with 10% BSA (bull serum albumin) at room temperature for 60 min to block nonspecific antibodies. Immunohistochemical staining was performed with rabbit anti-VEGFR2 antibody (Proteintech Group) or mouse anti-VTN (Vitronectin) antibody (Proteintech Group) respectively at room temperature for 2 h. After incubation with the corresponding secondary antibodies for 20 min, the bound complex was visualized by using the SuperPicTure polymer detection kit (No.87–8963; Invitrogen). To evaluate expressions of target genes, four representative areas were selected and were observed at 400× magnifcation respectively. The percentage of positive cells was assessed by counting the number of positive cells divided by all cancer cells under a selected area. The positive grades were determined as follows: 0, no positive tumor cells; 1, 1–10% positive tumor cells; 2, 11–50%; 3, 51–100%. The stating intensities were interpreted by the presence of yellow- or brown-colored cells in the target antigen sites, and were scored as follows: 0, no detectable signal; 1, mild staining –light yellow color; 2, moderate – yellow color; 3, intense – brown color. The final scores were obtained through multiplying the positive grade by the staining intensity score, leading to seven scores including 0, 1, 2, 3, 4, 6, and 9. Final scores ≤4 were regarded as low expression, while ≥6 were defined as high expression.

### Survival analysis

For analysis of survival data of 156 surgical cancer specimens, Kaplan–Meier curves were constructed, and statistical analysis was carried out using the log-rank test. Overall survival (OS) was defined as the time from beginning of surgery to death from any cause or the last date of follow-up. DFS (disease-free survival), or recurrence-free survival, is defined as the time from randomization to the first of either recurrence or relapse, second cancer, or death. Kaplan–Meier plotter (KM plotter, www.kmplot.com) was capable to assess the effect of 54,675 genes on survival using over 10,000 cancer samples. The 870 patients with gastric cancer were split into two groups according to the expression of a particular gene (high vs. low expression). The overall survival was analyzed using a Kaplan–Meier plot. The hazard ratio (HR) with 95% confidence intervals and log rank *P* value were calculated and displayed on the webpage.

### Cell culture and reagents

Human gastric cancer cell lines MKN-45, MKN-28, NCI-N87 and SCH and immortalized normal human gastric mucosal epithelial cell line GES-1 were obtained from American Type Culture Collection (ATCC). All cells were cultured in RPMI1640 medium (Invitrogen) with 10% fetal bovine serum (Invitrogen) and 37 °C 5% CO_2_. Apatinib was purchased from Hengrui Medicine Co. Ltd. (Jiangsu, China).

### Real-time PCR

Total RNA was extracted using TRIzol reagent (Invitrogen) according to the manufacturer’s protocol. After spectrophotometric quantification, 1 μg total RNA in a final volume of 20 μL was used for reverse transcription with PrimeScript RT Reagent kit (Takara, Otsu, Shiga, Japan) according to the manufacturer’s protocol. Aliquots of cDNA corresponding to equal amounts of RNA were used for quantification of mRNA by real-time PCR using the LightCycler 96 Real-time Quantitative PCR Detection system (Roche, Indianapolis, IN, USA). The reaction system (25 μL) contained the corresponding cDNA, forward and reverse primers, and SYBR Green PCR master mix (Roche). All data were analyzed using GAPDH gene expression as an internal standard. The specific primers are presented as follows: ①. VEGFR2 forward:5’-GGACTCTCTCTGCCTACCTCAC-3′, VEGFR2 reverse:5’-GGCTCTTTCGCTTACTGTTCTG-3′; ②. VTN forward:5’-TCACCAAGAGTCATGCAAGGG-3′, VTN reverse:5’-ACTCAGCCGTATAGTCTGTGC-3′; ③. GAPDH forward:5’-AGAAGGCTGGGGCTCATTTG-3′, GAPDH reverse:5’-AGGGGCCATCCACAGTCTTC-3′.

### Western blot

Total protein was extracted using a lysis buffer containing 50 mM Tris–HCl (pH 7.4), 150 mM NaCl, 1% Triton X-100, 0.1% SDS, 1 mM EDTA, and supplemented with protease inhibitor cocktail kit (Roche). The protein extract was loaded onto an SDS-polyacrylamide gel, size-fractionated by electrophoresis, and then transferred to polyvinylidene fluoride (PVDF) membranes (Bio-Rad Laboratories, CA, USA). After blocking in 5% non-fat milk for 1 h, the membranes were incubated overnight with primary antibodies at 4 °C. The protein expression was determined using horseradish peroxidase-conjugated antibodies followed by enhanced chemiluminescence (ECL, Millipore, St Charles, MO, USA) detection. The intensity of the bands was captured by JS-1035 image analysis scanning system (Peiqing Science & Technology, Shanghai, China). β-actin was used as the internal control.

### RNA interference and generation of stably knockdown cell lines

The sequences of small interfering RNA against human VEGFR2 (①. 5′-GCGGCTACCAGTCCGGATA-3′, ②. 5′-GGAAATCTCTTGCAAGCTA-3′) or VTN (①. 5’-GCAGACACCTGTTCTGAAA-3′, ②. 5’-GGAAGACCTACCTCTTCAA-3′) were cloned into a pGCL-EGFP plasmid (Genechem, Shanghai, China), which encodes an HIV-derived lentiviral vector containing a multiple cloning site for insertion of short hairpin RNA (shRNA) constructs to be driven by an upstream U6 promoter and a downstream CMV promoter–EGFP fluorescent protein. A negative control vector containing the sequence of 5’-TTCTCCGAACGTGTCACGT-3′ was used. Cells were infected with lentivirus produced by Genechem. Forty-eight hours later, EGFP positive cells were sorted by using flow cytometry and expanded for further experiments.

### Plasmids construction and generation of stably expressing cell lines

The coding sequences of VEGFR2 and VTN were amplified by PCR from homo cDNA using PrimerSTAR HS DNA polymerase (TAKARA, Otsu, Shiga, Japan), and the resulting PCR products were cloned into pcDNA3.1(+) (Invitrogen). All plasmid constructs were confirmed by sequencing. Cells were transfected with plasmids by Lipofectamine 3000 (Invitrogen) according to manufacturer’s protocol. Forty-eight hours later, the transfected cells were selected and maintained in medium containing 800 μg/ml G418 (Invitrogen) for 14 days. The drug resistant stably clones were isolated, collected and expanded for further experiments.

### Cell proliferation assay

Cell proliferation was measured by using Cell Counting Kit-8 (CCK-8) detection kit (Beyotime Biotechnology, China). Cells with a concentration of 5 × 10^3^ per well were seeded in 96-well plates. To evaluate the cytotoxicity of apatinib on cancer cells, different doses of apatinib were added to the culture. 24, 48, 72, 96 and 120 h later, 10 μL CCK-8 solution was added to each well and followed an incubation at 37 °C for 4 h. The absorbance in each well was measured at 450 nm using a microplate ELISA reader (Bio-Rad Laboratories, CA, USA). The relative cell viability was calculated as follows: relative absorbance = (mean absorbance at each time point/mean respective absorbance at 24 h).

### Invasion assay

A total of 100 μL of Matrigel (1:30 dilution in serum-free RPMI1640 medium) was added to each Transwell polycarbonate filter (8-μm pore size, Corning, NY, USA) and incubated with the filters at 37 °C for 6 h. Cells were trypsinized and washed three times with DMEM medium containing 1% FBS, followed by resuspention in RPMI1640 containing 1% FBS at a density of 2 × 10^6^ cells/ml. The cell suspensions (100 μL) were seeded into the upper chambers, and 600 mL of RPMI1640 medium containing 10% FBS were was added to the lower chambers. Cells (2 × 10^5^/well) were allowed to invade for 12 h and membranes were then stained with 1% methylrosanilinium chloride. Cells that had migrated to the underside of the filter were counted using a light microscope in five randomly selected fields.

### Nude mouse tumor xenograft model and treatment

Four-week-old female BALB/c athymic nude mice were purchased from Shanghai SLAC Laboratory Animal Co. Ltd. (Shanghai, China) and received humane care according to the Soochow University Institutional Animal Care and Treatment Committee. Cells were injected into the left flanks of the mice in a total volume of 100 μL (0.5 × 10^7^ cells). At the end of this experiment, the tumor tissues were dissected out, imaged, and weighed up. Apatinib was dissolved in DMSO and administered daily by intragastric administration at a dose of 10 mg/kg for 3 weeks.

### Microarray assay

Total RNA was extracted using TRIzol Reagent (Life technologies, Carlsbad, CA, US) according to the manufacturer’s instructions. RNA integrity was checked by an Agilent Bioanalyzer 2100. Gene expression was performed by Affymetrix GeneChip Human Genome U133 Plus 2.0 array platform. The hybridization occurred in a Hybridization Oven 645 (Affymetrix Inc.). The chips were then washed and stained in a Fluidics Station 450 (Affymetrix Inc.) and the arrays were scanned with a Gene Chip Scanner 3000 and Command Console Software 3.1 with default settings. The selection criterion was > 1.3-fold difference in expression (difference in upregulated expression > 1.3-fold; difference in downregulated expression < 0.77-fold). Hierarchical clustering of samples was performed using an average linkage algorithm using TIGR Multiexperiment Viewer (The Institute for Genomic Research, Rockville, MD, USA).

### Bioinformatic analysis

Gene ontology (GO) is a common method for annotating genes, gene products and sequences to underlying biological phenomena; the Kyoto Encyclopedia of Genes and Genomes (KEGG) is an integrated database resource for biological interpretation of genome sequences and other high-throughput data. Both GO and KEGG analyses were performed using the DAVID database (https://david.ncifcrf.gov/), which is a bioinformatics data resource composed of an integrated biology knowledge base and analysis tools to extract meaningful biological information from large quantities of genes and protein collections. Genetic networks and functional classification of differentially expressed genes were investigated with the ingenuity pathway analysis (IPA, Ingenuity Systems, Mountain View, CA, USA, http://www.ingenuity.com), a web delivered tool that enables the discovery, visualization, and exploration of molecular interaction networks in gene expression data.

### Statistical analysis

Data was represented as the mean ± SD and the difference among groups was evaluated by one- way ANONA, two-tailed Student’s T test. Repeated ANONA analysis was performed in analysing the difference in growth of implanted tumors between groups. All statistical analyses were performed using the SPSS 16.0 (Chicago, USA). A *p* value of < 0.05 was considered statistically significant.

## Results

### High expression of VEGFR2 in gastric cancer specimens was a poor prognostic factor

**Fig. 1:**
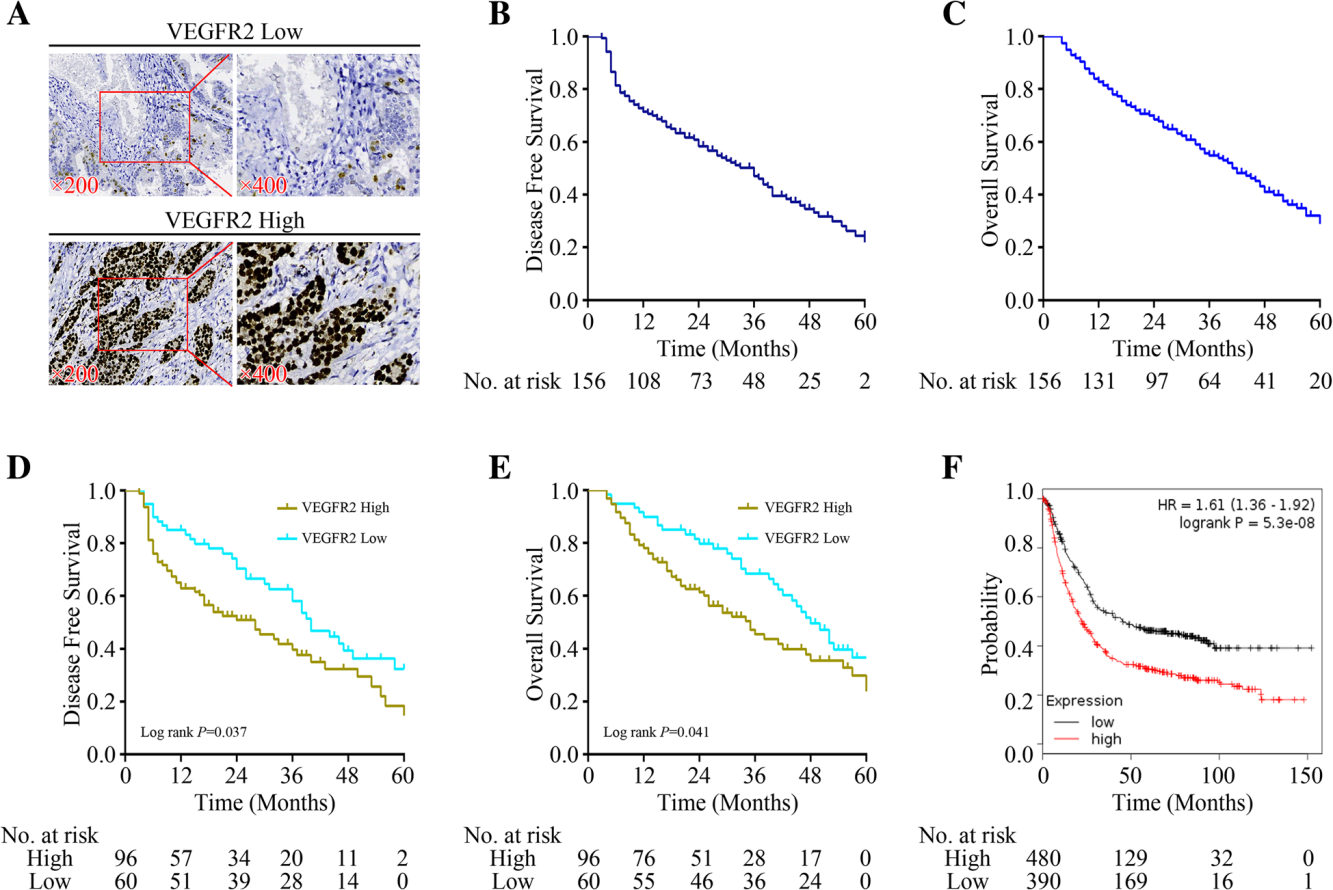
Correlationship between VEGFR2 expression in cancer specimens and survival of gastric cancer patients. **a** Representative images of anti-VEGFR2 staining in human gastric cancer tissues. **b** & **c** Disease-free survival (DFS) and Overall survival (OS) of 156 gastric cancer patients. **d** & **e** Disease-free survival (DFS) and Overall survival (OS) of 156 gastric cancer patients according the VEGFR2 status. **f** Overall survival (OS) of 870 gastric cancer patients according the VEGFR2 status using online Kaplan-Meier Plotter database

To determine the potential role of VEGFR2 in the progression and metastasis of gastric cancer, 156 human gastric cancer specimens were collected and the levels of VEGFR2 expression were characterized by immunohistochemistry. High VEGFR2 were detected in the nucleus or both in nucleus and cytoplasm of cancer cells (Fig. 1a). The relationship between VEGFR2 and survival in 156 gastric cancer patients was analysed by Kaplan-Meier method. Median overall survival (OS) and disease-free survival (DFS) were 42 and 36 months respectively (Fig. 1b & c). High expression of VEGF2 was significantly correlated with poorer DFS and OS (*P* = 0.037 and 0.041) (Fig. 1d & e). To evaluate the correlation in a larger sample size in gastric cancer, we employed online Kaplan-Meier Plotter tool and found more convincing results that high VEGFR2 was a significant prognostic factor for poorer OS *(P* = 5.3e-08, HR 1.61, 95% CI 1.36–1.92) (Fig. 1f). Besides, we also analyzed the relationship between the expressions of VEGFR2 and VTN with clinicopathological features. As shown in Table 1, There was no significant relationship between VEGFR2 or VTN expression and gender (*P* = 0.331), age(*P* = 0.427), Tumor size (*P* = 0.153), Lauren type(*P* = 0.739) and tumor differentiation(*P* = 0.912),but both of the two genes were colsely related with T staging, N staging and disease stage (*P* < 0.01).

**Table 1:** Relationship between the expressions of VEGFR2 and VTN with clinicopathological features

Clinicopathologic features	No.	VEGFR2 expression				VTN expression			
		High (No.)	Low (No.)	χ2	*P* value	High (No.)	Low (No.)	χ2	*P* value
Gender		96	60			92	64		
Men	110	65	45	0.944	0.331	62	48	1.051	0.305
Women	46	31	15			30	16		
Age (years)									
<65	90	53	37	0.631	0.427	51	39	0.468	0.494
≥65	66	43	23			41	25		
Tumor size (cm)									
<5	101	58	43	2.047	0.153	55	46	2.418	0.12
≥5	55	38	17			37	18		
Lauren type									
Intestinal type	91	55	36	0.111	0.739	53	38	0.048	0.826
Diffuse type	65	41	24			39	26		
Degree of differentiation									
Highly	46	28	18	0.012	0.912	26	20	0.162	0.687
Moderately and Poorly	110	68	42			66	44		
Depth of invasion									
T1, T2	61	28	33	10.348	<0.001^**^	27	34	8.961	<0.003^**^
T3, T4	95	68	27			65	30		
Lymphonodus metastasis									
N0, N1	50	22	28	9.563	<0.002^**^	21	29	8.763	<0.003^**^
N2, N3	106	74	32			71	35		
AJCC stage									
I, II	51	18	33	22.05	<0.001^**^	18	33	17.561	<0.001^**^
III, IV	105	78	27			74	31		

^**^indicates *P<0.01*

### VEGFR2 promoted the growth and metastasis of gastric cancer cells

**Fig. 2:**
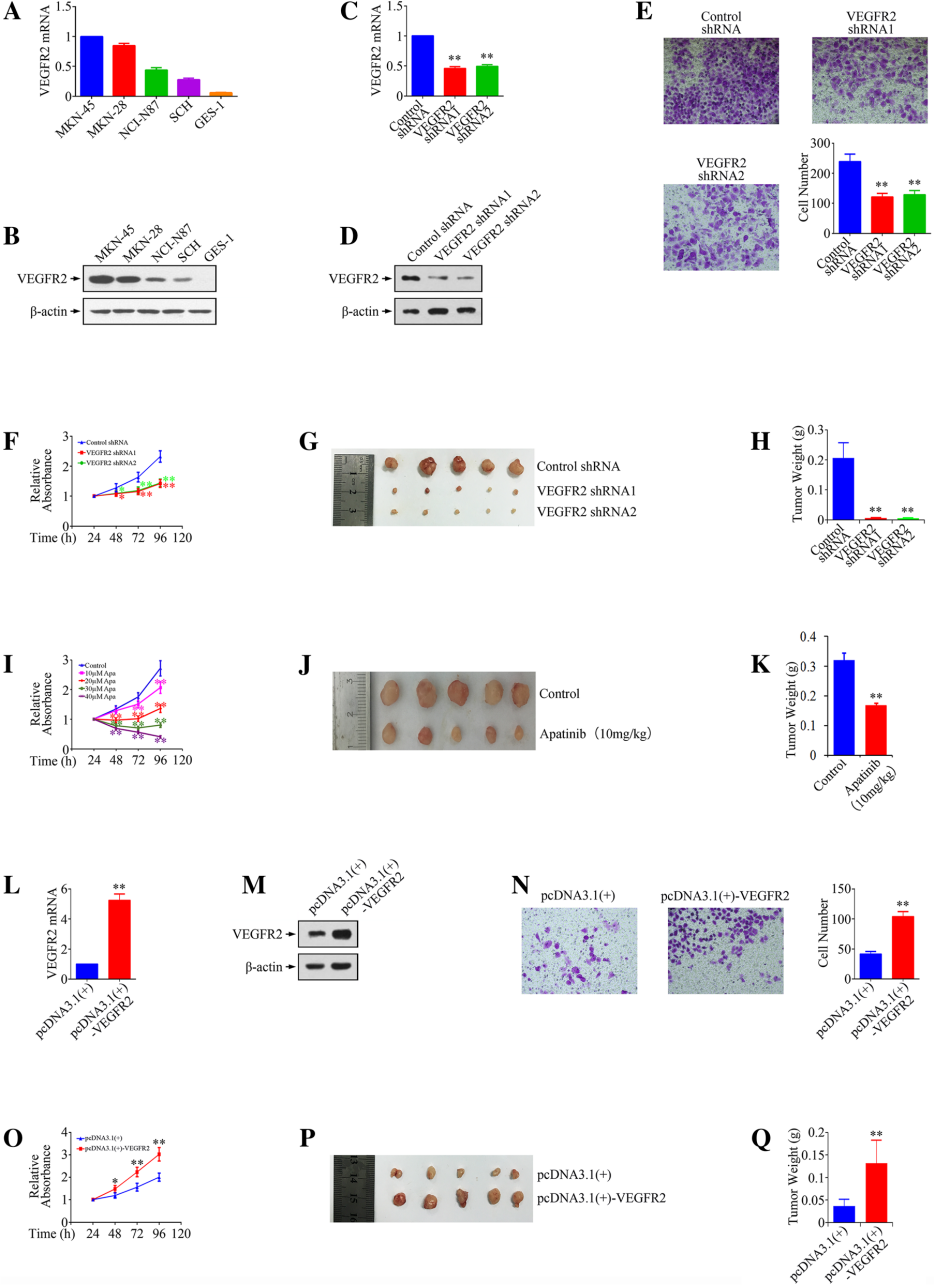
Modulation of VEGFR2 expression changes the growth and metastasis of gastric cancer cells. **a** & **b** Real-time PCR and Western blot analysis of relative levels of VEGFR2 in gastric cancer cell lines and GES-1 cells. **c** & **d** Confirmation of VEGFR2 knockdown in MKN-45 cells by using Real-time PCR and Western blot analysis. **e** The effect of VEFR2 silencing on the invasion of MKN-45 cells. **f** The effect of VEFR2 knockdown on the growth of MKN-45 cells in vitro. **g** Optical images of MKN-45 xenograft tumors. Tumors were dissected out 20 days after cell injection. **h** Tumor weight of MKN-45 xenograft tumors. **i** The effect of different doses of Apatinib (Apa) on the growth of MKN-45 cells in vitro. **j** Optical images of MKN-45 xenograft tumors after treatment with Apatinib (10 mg/kg). **k** Tumor weight of MKN-45 xenograft tumors after treatment with Apatinib. **l** & **m** Confirmation of VEGFR2 overexpression in SCH cells by using Real-time PCR and Western blot analysis. **n** The effect of VEFR2 overexpression on the invasion of SCH cells. **o** The effect of VEFR2 overexpression on the growth of SCH cells in vitro. **p** Optical images of inoculated SCH tumors. Tumors were dissected out 20 days after cell injection. **q** Tumor weight of inoculated SCH tumors. *P < 0.05, **P < 0.01, significant differences vs. the respective control groups

To explore the role of VEGFR2 in gastric cancer pathogenesis, we firstly characterized VEGFR2 in gastric cancer cell lines. We screened a panel of four gastric cancer cell lines (MKN-45, MKN-28, NCI-N87 and SCH) and immortalized normal human gastric mucosal epithelial cell line GES-1 for expressions of VEGFR2 mRNA and protein using real-time PCR and Western blot, respectively. We found that four gastric cancer cell lines had detectable expression of VEGFR2 mRNA and protein, but lack of VEGFR2 expression in GES-1 (Fig. 2a & b). To determine whether VEGFR2 expression is required for gastric cancer growth and metastasis, we knocked down VEGFR2 with specific shRNAs in MKN-45 cells (Fig. 2c & d), which have a high level of VEGFR2, and overexpressed VEGFR2 by cDNA construct transfection in SCH cells (Fig. 2l & m), which have a low level of VEGFR2. By using matrigel transwell invasion assay, we found out that down-regulation of VEGFR2 resulted in decreased cell invasive ability (Fig. 2 e), while overexpression of VEGFR2 promoted in vitro invasion (Fig. 2n). Moreover, knockdown of VEGFR2 repressed proliferation, while overexpression of VEGFR2 accelerated growth of gastric cancer cells both in vitro (Fig. 2 f & o) and in vivo (Fig. 2 g, h, p & q). In addition, we used Apatinib (an antiangiogenic agent targeting the intracellular ATP-binding site of vascular endothelial growth factor receptor 2 (VEGFR2)) to confirm the effect of VEGFR2 inhibitor on angiogenesis independent tumorigenesis. As shown in Fig. 2 i, Apatinib inhibited MKN-45 cells proliferation in both dose- and time-dependent manners. Besides, Apatinib repressed the growth of gastric cancer subcutaneous xenografts remarkably, indicating that VEGFR2 inhibitor play an effective anti-cancer role in vivo (Fig. 2 j & k).

### Identification of key genes at the downstream of VEGFR2 pathways in gastric cancer

**Fig. 3:**
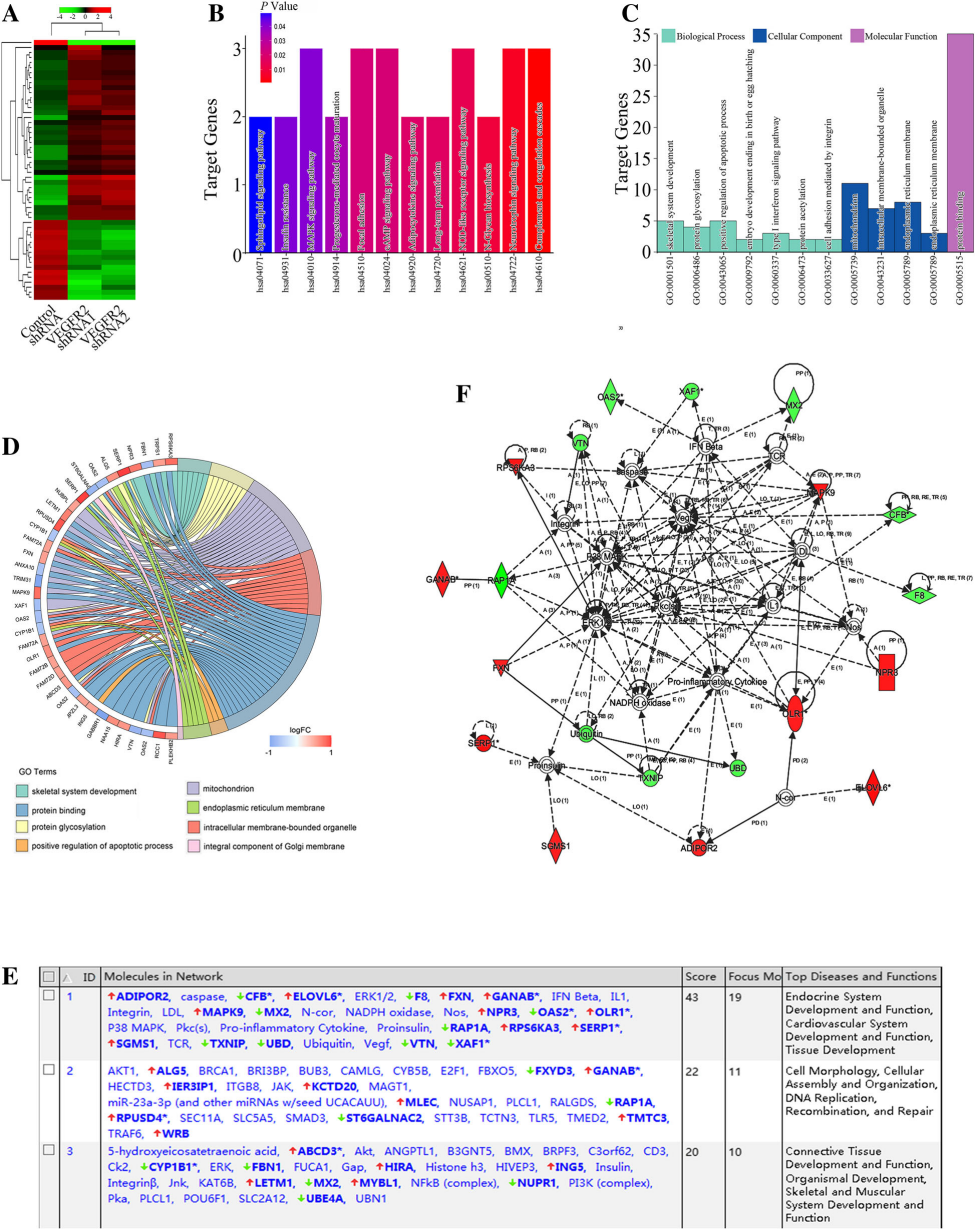
Microarray and bioinformatic analysis of VEGFR2 signaling in gastric cancer cells. **a** Two-dimensional hierarchical clustering image of overall genes differentially regulated by VEGFR2 in MKN-45 gastric cancer cells. **b** KEGG pathway analysis of differentially expressed genes (DEGs). **c** GO analysis of DEGs. **d** Detail GO categories related to DEGs. **e** Top scored three networks with their respective scores obtained using the ingenuity pathway analysis (IPA). **f** The highest rated networks in IPA. Upregulated genes are depicted in red, whereas downregulated genes are depicted in green

To gain further insight into the functional roles of VEGFR2 in biological functions in gastric cancer, we evaluated mRNA expression profiling after VEGFR2 knockdown by using microarray. Microarray data were uploaded as Additional file 1. Clustering data of up- and down-modulated mRNAs were illustrated in Fig. 3a. After down-regulating of VEGFR2 in gastric cancer, we identified 56 differentially expressed genes (DEGs), including 37 up-regulated genes and 19 down-regulated genes. To evaluate the functional roles of VEGFR2-regulated genes, pathway analysis was used and target genes modulated by VGEFR2-shRNAs in gastric cancer were significantly enriched in multiple KEGG pathways, represented by MAPK, focal adhesion and cAMP signaling pathways (Fig. 3g). We next performed gene ontology (GO) analysis on all target genes which were classified into three categories: [1]. biological process, including skeletal system development, protein glycosylation, positive regulation of apoptosis process, embryo development ending in birth, type I interferon signaling pathway, protein acetylation and cell adhesion mediated by integrin; [2]. cellular component, including mitochondrion, intracellular membrane-bounded organelle, endoplasmic reticulum membrane and endoplasmic reticulum membrane; [3]. molecular function, including protein binding (Fig. 3b c &d). Figure 3e showed the top scored three networks with their respective scores obtained using the ingenuity pathway analysis (IPA). Individual relationship between DEGs and GO subtypes were presented in Fig. 3f.

The DEGs were further subjected to the ingenuity pathway analysis (IPA) to explore the potential molecular interaction networks. The top scored network concerned Endocrine System Development and Function, Cardiovascular System Development and Function, as well as Tissue Development (Fig. 3b c &d). Among the 56 DEGs, 19 ones were involved in the top scored network.

### Target genes of VEGFR2 as prognostic factors in gastric cancer

**Fig. 4:**
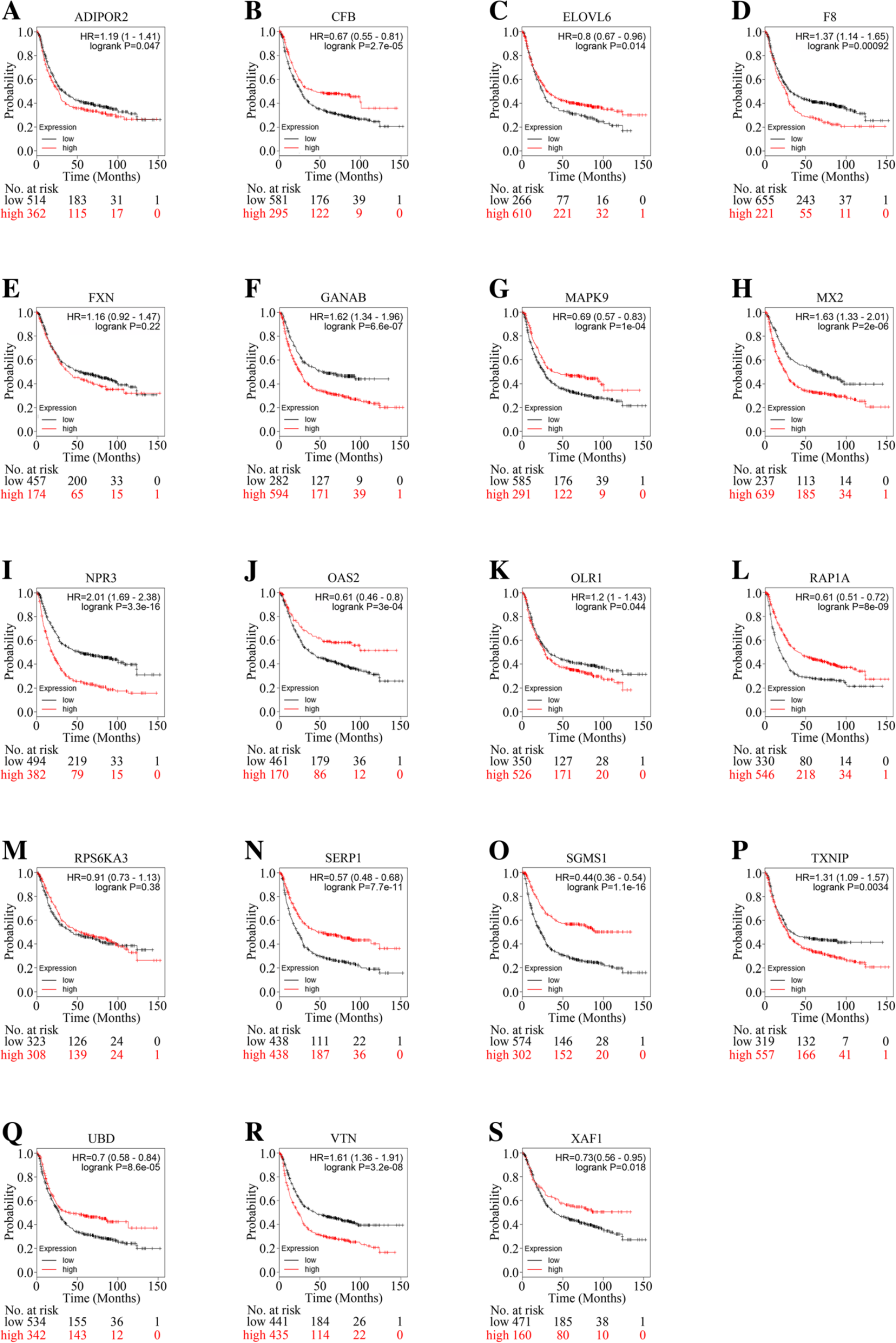
Prognostic value of DEGs in gastric cancer patients by using online Kaplan-Meier Plotter analysis. **a** Overall survival (OS) of 876 gastric cancer patients according the ADIPOR2 status using online Kaplan-Meier Plotter database. **b** Overall survival (OS) of 876 gastric cancer patients according the CFB status using online Kaplan-Meier Plotter database. **c** Overall survival (OS) of 876 gastric cancer patients according the ELOVL6 status using online Kaplan-Meier Plotter database. **d** Overall survival (OS) of 876 gastric cancer patients according the F8 status using online Kaplan-Meier Plotter database. **e** Overall survival (OS) of 631 gastric cancer patients according the FXN status using online Kaplan-Meier Plotter database. **f** Overall survival (OS) of 876 gastric cancer patients according the GANAB status using online Kaplan-Meier Plotter database. **g** Overall survival (OS) of 876 gastric cancer patients according the MAPK9 status using online Kaplan-Meier Plotter database. **h** Overall survival (OS) of 876 gastric cancer patients according the MX2 status using online Kaplan-Meier Plotter database. **i** Overall survival (OS) of 876 gastric cancer patients according the NPR3 status using online Kaplan-Meier Plotter database. **j** Overall survival (OS) of 631 gastric cancer patients according the OAS2 status using online Kaplan-Meier Plotter database. **k** Overall survival (OS) of 876 gastric cancer patients according the OLR1 status using online Kaplan-Meier Plotter database. **l** Overall survival (OS) of 876 gastric cancer patients according the RAP1A status using online Kaplan-Meier Plotter database. **m** Overall survival (OS) of 631 gastric cancer patients according the RPS6KA3 status using online Kaplan-Meier Plotter database. **n** Overall survival (OS) of 876 gastric cancer patients according the SERP1 status using online Kaplan-Meier Plotter database. **o** Overall survival (OS) of 876 gastric cancer patients according the SGMS1 status using online Kaplan-Meier Plotter database. **p** Overall survival (OS) of 876 gastric cancer patients according the TXNIP status using online Kaplan-Meier Plotter database. **q** Overall survival (OS) of 876 gastric cancer patients according the UBD status using online Kaplan-Meier Plotter database. **r** Overall survival (OS) of 876 gastric cancer patients according the VTN status using online Kaplan-Meier Plotter database. **s** Overall survival (OS) of 631 gastric cancer patients according the XAF1 status using online Kaplan-Meier Plotter database

To further clarify whether the 19 target genes of top scored network in IPA could act as prognostic indicators in gastric cancer, online Kaplan-Meier Plotter tool was utilized (Fig. 4a-s). VTN (Fig. 4r), MX2 (Fig. 4h), F8 (Fig. 4d) and TXNIP (Fig. 4p), which could be down-regulated by VEGFR2-knockdown, were associated with poorer survival. While OAS2 (Fig. 4j), XAF1 (Fig. 4s), CFB(Fig. 4b), RAP1A (Fig. 4l), and UBD (Fig. 4q), which could also be down-regulated by VEGFR2-knockdown, predicted better survival. Among the up-regulated genes after VEGFR2-knockdown, ELOVL6 (Fig. 4c), MAPK9 (Fig. 4g), SERP1 (Fig. 4n) and SGMS1 (Fig. 4o) presented to be good prognostic indicators.While ADIPOR2 (Fig. 4a), GANAB (Fig. 4f), NPR3 (Fig. 4i), and OLR1 (Fig. 4k), which could also be upregulated by VEGFR2-knockdown, predicted poorer survival. FXN (Fig. 4e) and RPS6KA3 (Fig. 4m) were not statistically significant.Therefore, the pro-angiogenic effect of VEGFR2 may be executed through mechanisms involving multiple angiogenesis-related genes.

### VEGFR2 acted as a upstream regulator of VTN in gastric cancer

**Fig. 5:**
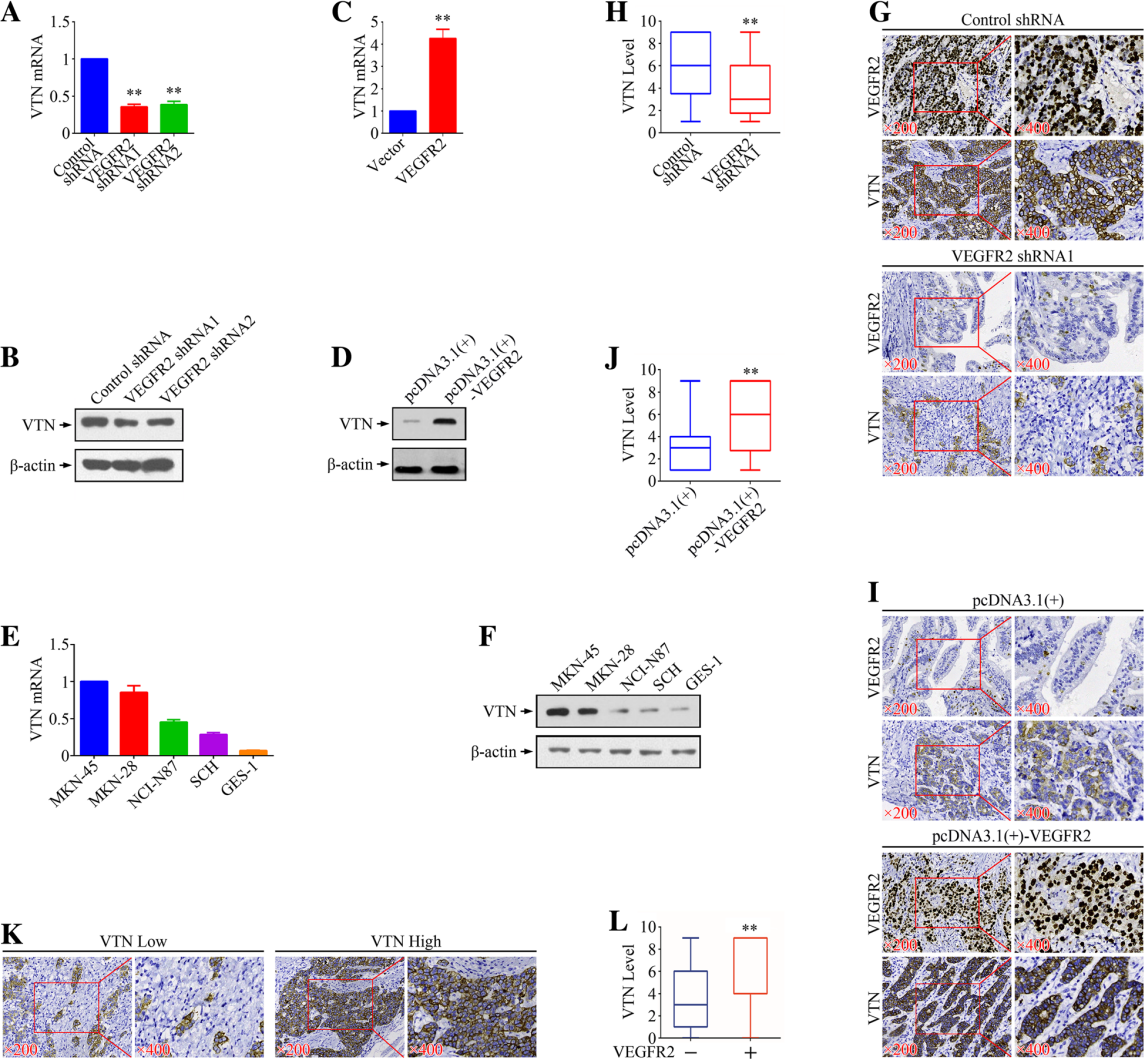
The effect of VEGFR2 on VTN expression. **a** & **b** Real-time PCR and Western blot analysis of relative levels of VTN in MKN-45 cells after VEGFR2 knockdown. **c** & **d** Real-time PCR and Western blot analysis of relative levels of VTN in SCH cells after VEGFR2 overexpression. **e** & **f** Real-time PCR and Western blot analysis of relative levels of VTN in a panel of gastric cancer cell lines and immortalized normal human gastric mucosal epithelial cell line GES-1. **g** & **h** IHC staining analysis of VTN in MKN-45 xenograft tumors. **i & j** IHC staining analysis of VTN in SCH xenograft tumors. **k** Representative images of anti-VTN staining in human gastric cancer tissues. **l** Relationship between expressions of VEGFR2 and VTN in human gastric cancer tissues. **P < 0.01, significant differences vs. the respective control groups

We next focused on the relationship between VEGFR2 and VTN. When knocked down by VEGFR2 shRNAs, MKN-45 cell line represented decreased VTN levels (Fig. 5a & b). In SCH cells, overexpression of VEFGR2 could induced the up-regulation of VTN (Fig. 5c & d). We then evaluated expressions of VTN in gastric cancer cell lines and identified a similar expression trends as VEGFR2 in both protein and mRNA levels (Fig. 5e & f). In xenograft models, knockdown of VEGFR2 inhibited VTN expression while overexpression of VEGFR2 promoted the expression of VTN (Fig. 5g-j). In 156 gastric cancer specimens mentioned above, VEGFR2 was found positively correlated to VTN in immunohistochemistry assays (Fig. 5k & l). Therefore, VTN presented to be a downstream of VEGFR2.

### VTN promoted the growth and metastasis of gastric cancer cells

**Fig. 6:**
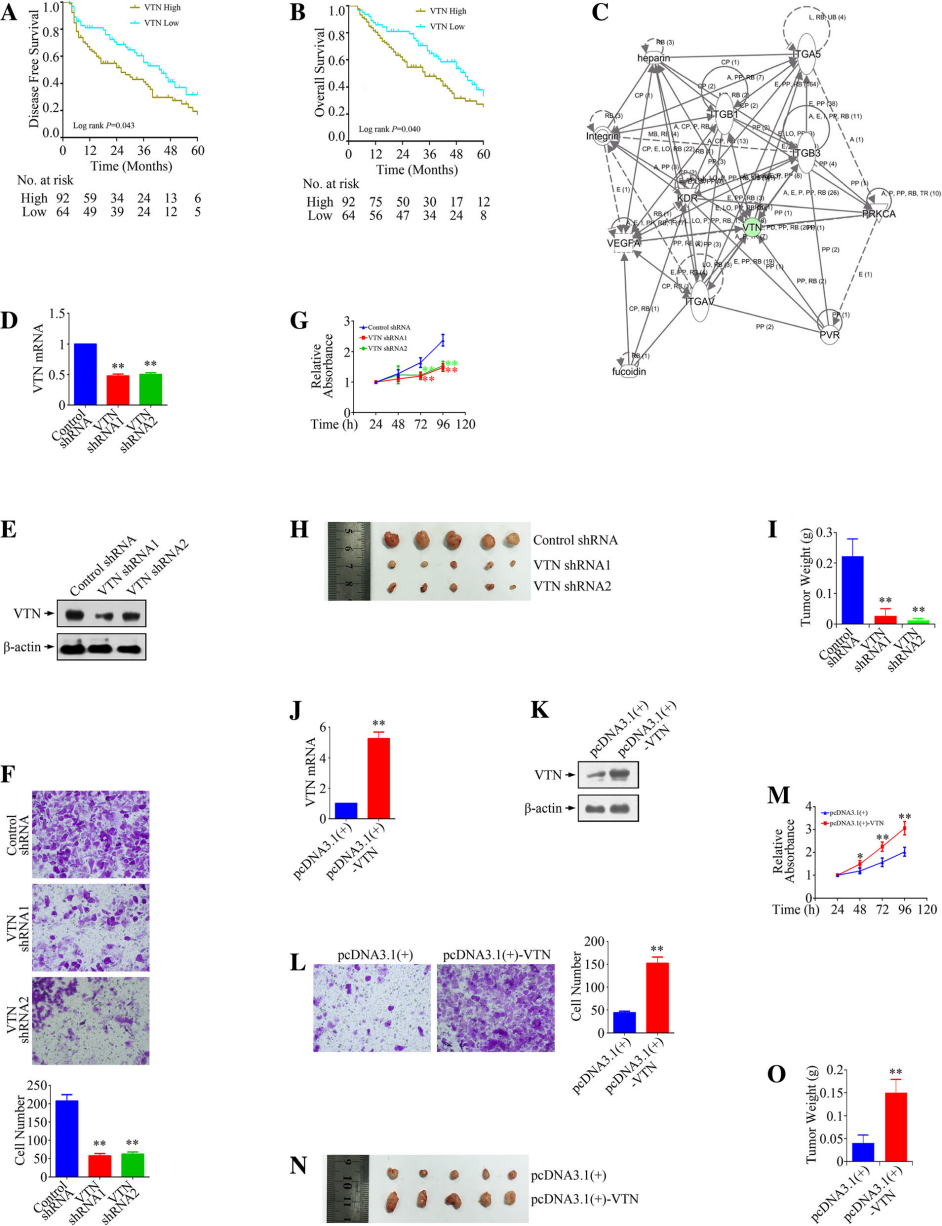
The effect of VTN on the growth and metastasis of gastric cancer cells. **a** & **b** Disease-free survival (DFS) and Overall survival (OS) of 156 gastric cancer patients according the VTN status. **c** Analysis of network regarding to VTN using IPA. **d** & **e** Confirmation of VTN knockdown in MKN-45 cells by using Real-time PCR and Western blot analysis. **f** The effect of VTN silencing on the invasion of MKN-45 cells. **g** The effect of VTN knockdown on the growth of MKN-45 cells in vitro. **h** Optical images of MKN-45 xenograft tumors. Tumors were dissected out 20 days after cell injection. **i** Tumor weight of MKN-45 xenograft tumors. **j** & **k** Confirmation of VTN overexpression in SCH cells by using Real-time PCR and Western blot analysis. **l** The effect of VTN overexpression on the invasion of SCH cells. **m** The effect of VTN overexpression on the growth of SCH cells in vitro. **n** Optical images of inoculated SCH tumors. Tumors were dissected out 20 days after cell injection. **o** Tumor weight of inoculated SCH tumors. *P < 0.05, **P < 0.01, significant differences vs. the respective control groups

To confirm whether VTN could be a pro-oncogenic executor, we investigated the relationship between VTN expression and biological function of gastric cancer cells. In 156 gastric cancer patients (Fig. 6a & b), VTN act as a poor prognostic factor both for disease-free survival and overall survival (*P* = 0.043 and 0.040), in consisting with the data obtained from Kaplan-Meier Plotter datasets. Ingenuity pathway analysis (IPA) suggested the interaction between VTN and members of intergrin (Fig. 6c). Figure 6 d & e confirmed the VTN knockdown in MKN-45 cells, while Fig. 6j & k confirmed the VTN overexpression in SCH cells by using Real-time PCR and Western blot analysis. Invasion of gastric cancer cells could be repressed by knockdown but could be accelerated by overexpression of VTN (Fig. 6f & l). Moreover, both in vitro and in vivo growth of gastric cancer cells could be inhibited by knockdown but could be promoted by overexpression of VTN (Fig. 6 g-i, m-o). Therefore, VTN presented to be an oncogene which located at the downstream of VEGFR2.

## Discussion

The regulation of VEGFR2 has been a topic of interest for numerous investigators in tumor pathophysiology, given its importance in the tumorigenesis and development of multiple cancers, including gastric cancer. It has been well-established that the expression of VRGFR2, and its ligands, was correlated with a poor prognosis, and the application of VEGFR2 targeted therapies, inhibiting the tumor related angiogenesis, has improved the outcomes of gastric cancer patients at advanced stages [3]. In breast cancer, VEGFR2 was only detected in the most aggressive subtype, triple negative breast cancer, leading to enhanced EMT (epithelial-mesenchymal transition) process and activation of NF-κB and β-catenin signaling pathways [8]. In breast cancer-initiating stem cells, when stimulated by VEGF, VEGFR2 binds to JAK2 and activates STAT3, and maintains cell self-renewal by promoting MYC and SOX2 expression and induces sphere formation [9]. Besides, in ovarian caner, VEGFR2 also mediates stem cell ability by activation of Src, which increased DNMT3A for miR-128-2 methylation and upregulated Bmi for stem-like cell proliferation [14]. Interestingly, VEGFR2 could also act as the downstream driver gene of circRNA-MYLK when binding to miR-29a and relieving its suppression for VEGFA, and promote Ras/ERK signaling pathway in bladder cancer [15]. Loss of PTEN and activated PI3K/AKT/mTOR are required for up-regulation of VEGFR2, and then activating NF-κB-dependent transcriptional activity to induce the loss of sensitivity of chemotherapy in glioma [17].

With respect to targeted therapy to VEGFR2 by targeting the cancer cells directly, VEGFR2 and its company STAT3 are inhibited by Apatinib and BCL-2 was consequently supressed, inducing autophagy and apoptosis eventually, implying the potential benefits when combing Apatinib and autophagy inhibitors in treatment of osteosarcoma [16]. However, the involvement of VEGFR2, the target of Apatinib, on the tumorigenesis and metastasis and the molecular mechanisms underlying the regulatory effects in gastric cancer cells remains elucidated.

In this study, we characterized the specific roles and functions of VEGFR2 in gastric cancer pathogenesis, not on the vascular compartment but on the whole cancer cell system. We first characterized VEGFR2 expression in primary gastric cancer tissues and found that level of VEGFR2 expression in gastric cancer were associated with poor survival in subjects from our institution and online database population. VEGFR2 promoted gastric cancer cell proliferation and invasion in vitro, and accelerated tumor growth in vivo. Furthermore, by high-throughput analysis, we predicted its regulatory pathway and delineated the downstream molecules and their relevance for survival. The target signaling pathway, such as MAPK and focal adhesion pathways, as well as target molecules, including VTN, were validated. These findings supported the angiogenesis independent manner of VEGFR2 functioning as a carcinogenic factor in gastric cancer.

VTN (vitronectin), as a cell-adhesion glycoprotein, is primarily localized in the ECM (extracellular matrix) and provides the facility to help tumor cells to breach through to the basement membrane in the process of cancer cell invasion [18]. Besides of ability to affect cancer cell adhesion, motility and invasion, VTN also assists cancer cells to resist the cell death induced by apoptotic induction [19]. In drug resistant multiple myeloma cells, the adhesion signaling molecules, such as VTN, was upregulated by Notch signaling pathway and conferred cell protection from drug induced apoptosis [20]. In nasopharyngeal carcinoma, VTN was identified as once of BPIFB1-interacting proteins and could be reduced by BPIFB1, leading to less formation of VTN-integrin αV complex, suppressing the EMT process, and inhibition of the activation of downstream FAK/Src/ERK signaling pathway [21]. However, in angiogenic cascade, the engagement of VTN and its receptor integrin αVβ3 needs the activation of Src, serving as a upstream factor, to activate the VEGFR2 signaling for endothelial cell adhesion and migration [22]. In the cancer development from the early stages to the advanced stages, VTN can be detected in up-regulated levels in a large scale clinical proteomics study in metastatic colorectal cancer, indicating its pro-invasion potential [23]. Intriguingly, VTN can also be detected to be secreted to blood serum in prostate cancer patients and act as circulating biomarker when combined with PSA (prostate-specific antigen) for early diagnosis of prostate cancer [24]. With respect of association of gastric cancer and VTN, only one document revealed that VTN acted as the downstream molecule of CEP65, which may provide gastric cancer cells with capacity to detach from the ECM, decreasing cell adhesion [25]. To our knowledge, for the first time, our results indicate that VTN also acts as the downstream of VEGFR2 pathway in gastric cancer tissues and as a poor prognostic factor in gastric cancer patients. Hence, VEGFR2 and VTN expression in cancer tissues may also serve as biomarkers for tumorigenesis and metastasis of gastric cancer and as direct therapeutic targets in cancer cell level for traditional anti-angiogenesis treatment.

In summary, our data indicated that higher levels of VEGFR2 and its target VTN in cancer specimens were associated with aggressiveness and poorer prognosis of gastric cancer. VEGFR2 promoted the proliferation and invasion, and provided the capacities of tumor formation in xenograft models in gastric cancer, which may be associated with downstream VTN. In addition, our data also suggests that multiple predicted target genes of VEGFR2 in co-expression network may act as prognostic factors in bioinformatic analysis with a larger sample of gastric cancer. VEGFR2/VTN axis in gastric cancer cells may be involved in tumorigenesis and metastasis in a pro-angiogenic-independent way although the precise mechanisms need to be further elucidated. Nevertheless, our study may provide a new and valuable target for design of therapies for intervention and a new cognitive perspective for the anti-angiogenesis therapies.

## Additional file

Additional file 1: The data of microarray analysis of VEGFR2 signaling in gastric cancer cells. (XLSX 18770 kb)
